# Supplementary material for: The Influence on Contaminant Bioavailability and Microbial Abundance of Lake Hongze by the South-to-North Water Diversion Project
Source: Int J Environ Res Public Health. 2019 Aug 23;16(17):3068. doi: 10.3390/ijerph16173068 (PMC6747398; doi:10.3390/ijerph16173068)
Supplement: Supplementary file 1 [file ijerph-16-03068-s001.zip › ijerph-560469-suppl xml.pdf]

International Journal of
Environmental Research and Public Health

Supplementary Materials: The Influence on Contaminants Bioavailability and Microbial Abundance of Lake Hongze by the South-to-North Water Diversion Project

Yu Yao, Peifang Wang and Chao Wang,

1. The DGT Preparation and Assembly Sequence

The one-dimensional distribution of 3-mercaptopropyl-functionalized silica DGT (diffusive gradients in thin films technique) and ZrO-Chelex DGT and the two-dimensional distribution of ZrO-DGT and AgI DGT were applied for the labile concentrations of Hg, As, Fe, Mg, Cd, Pb, Mn, P, as well as S.

1.1. 3-Mercaptopropyl Functionalized Silica Gel DGT

The diffusion gel was prepared with 15% acrylamide and a 0.3% agarose-derived cross-linker, as described by Zhang and Davison (1995). The resin gel was impregnated with 1.0 g of 3-mercaptopropyl functionalized silica gel (Sigma, San Francisco, CA, USA) in 10 mL of gel solution, which was the same as that used in the polyacrylamide diffusion gel. A schematic illustration of the DGT probe is shown in Figure S1 in the Supplementary Information. From bottom to top, the DGT device components consist of a plastic base, a Zr-oxide resin gel, a 0.40 mm thick diffusion gel, a 0.13 mm thick cellulose nitrate filter membrane (Whatman, Nasdaq, MN, USA, 0.45 mm pore size), and a plastic cover plate. The recovery of 3-mercaptopropyl functionalized silica gel DGT was 81% (RSD < 5%); this value was later applied to correct the results of accumulation by the gel.

1.2. Chelex DGT Probe for Fe, Mg, Cd, Pb, and Mn

The manufacturing process for ZrO-Chelex DGT was similar to that of the 3-mercaptopropyl functionalized silica gel DGT, except that the resin gels have the same components and assembly sequence as the DGT probes. The resin gel of the ZrO-Chelex DGT was impregnated with 2 g $ZrOCl_2{\cdot}8H_2O$ (Sigma, USA) and 1.0 g Chelex-100 (Bio-rad., Hercules, CA, USA) in 10 mL of gel solution. The recovery of ZrO-Chelex DGT was 87% (RSD < 5%); this value was later applied to correct the results of accumulation by the gel.

1.3. AgI DGT for Two-Dimensional Distribution of Labile S

The production process of the AgI resin gel was similar to that of the Chelex. The resin gel of the AgI DGT was impregnated with 0.75 mL AgI in 10 mL of gel solution. The fines of AgI that emerged on the surface of the resin gel were formed by the sequence mixture of $AgNO_3$ and KI solutions. The recovery of AgI DGT was 86% (RSD < 5%); this value was later applied to correct the results of accumulation by the gel.

1.4. ZrO–DGT for Two-Dimensional Distribution of Labile P

The resin gel of the ZrO-DGT was impregnated with 2 g $ZrOCl_2{\cdot}8H_2O$ (Sigma, USA) in 10 mL of gel solution. The fines of $ZrOCl_2.8H_2O$ that emerged on the surface of the resin gel were formed by the sequence mixture of $AgNO_3$ and KI solutions. The recovery of AgI DGT was 86% (RSD < 5%); this value was later applied to correct the results of accumulation by the gel.

2. The Details of the ex Situ Measurements

Dissolved total carbon (TOC-VCPH, Shimadzu, Columbia, MD, USA) and total nitrogen (Kjeltec TM 2300, Foss, Hilleroed, Denmark) were analyzed according to standard methods. Moisture content was measured through weighing the samples before and after oven-drying at 105 °C for over 16 h. The total concentrations of K and Na in sediments were obtained by the microwave digestion instrument, then analyzed by the atomic absorption spectrophotometer (TAS-990, Pgeneral, Beijing, China).

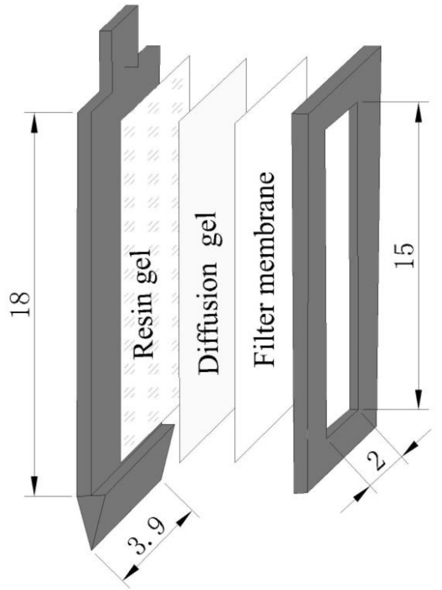

Figure S1. Schematic of DGT probe. To ensure holistic analysis of the labile elements in sediment under different conditions, the DGT probes should be inserted vertically into the sediment surface. At 2 cm distance of the top of the device was a marker label to ensure that the sediment–water interface was accurately identified during deployment.

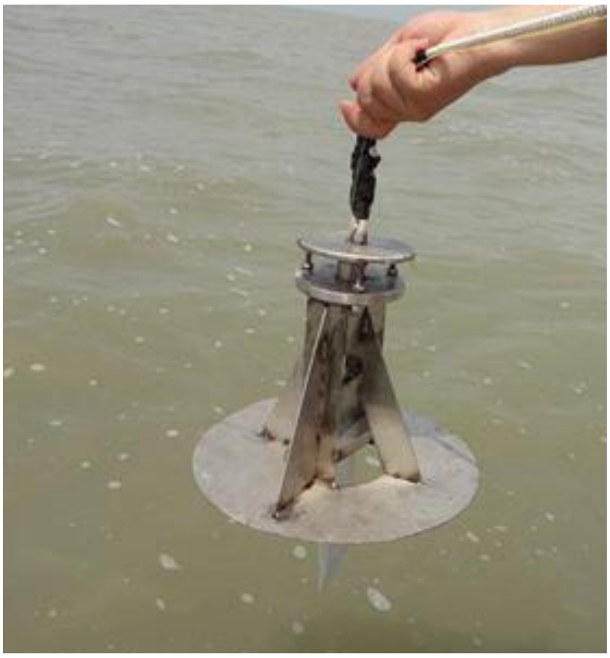

Figure S2. DGT release device. The DGT probe was deployed in the center of the device by a string, and the gravitational effects of the device could lead the probe inserted in the sediment. A buoy was applied as the label for the convenience of DGT retrieval.

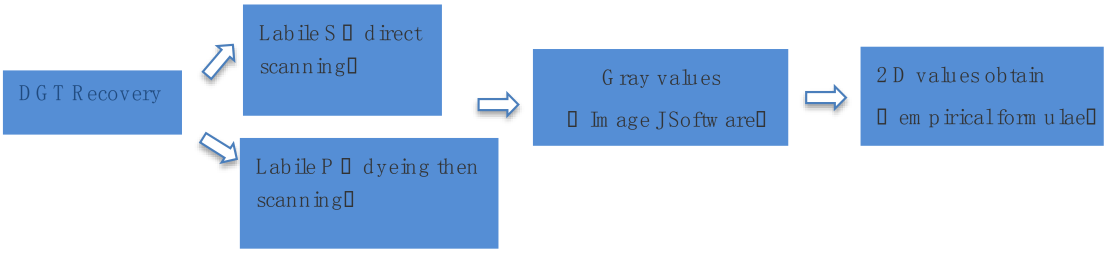

Figure S3. The computer-imaging densitometry (CID) operation process of labile S and P.

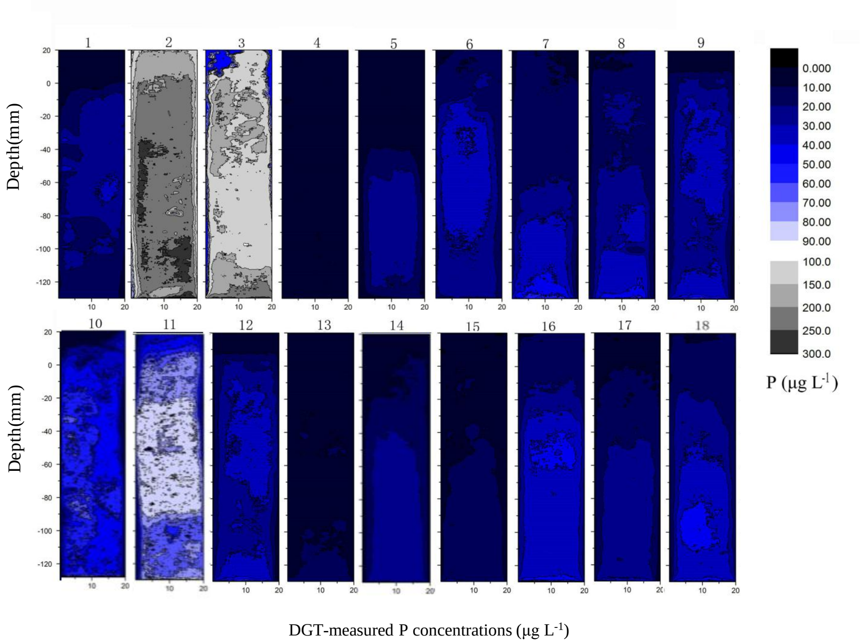

Figure S4. The DGT measured P concentrations of sampling sites in Lake Hongze.

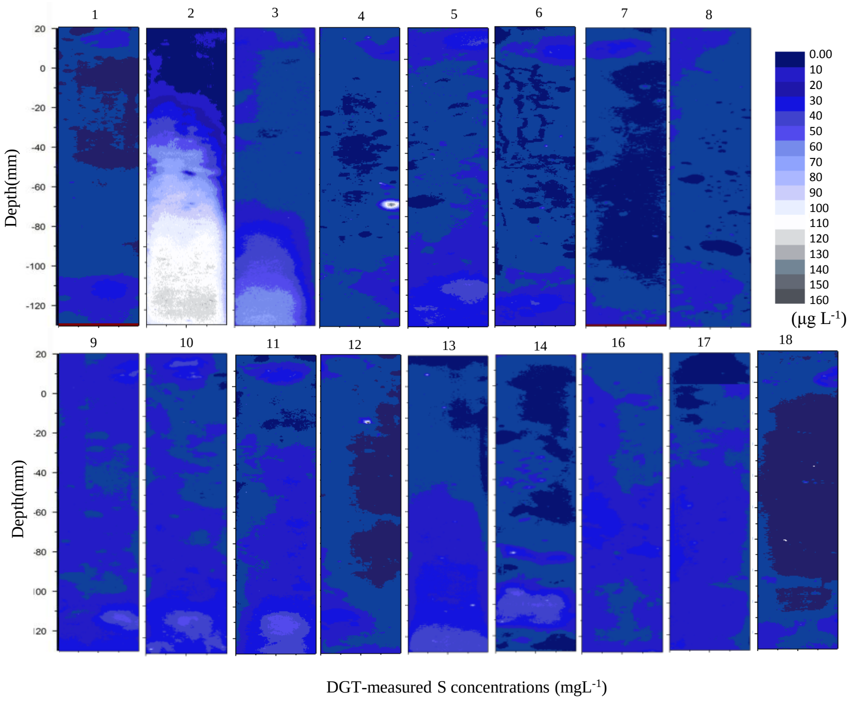

Figure S5. The DGT measured S concentrations of sampling sites in Lake Hongze.

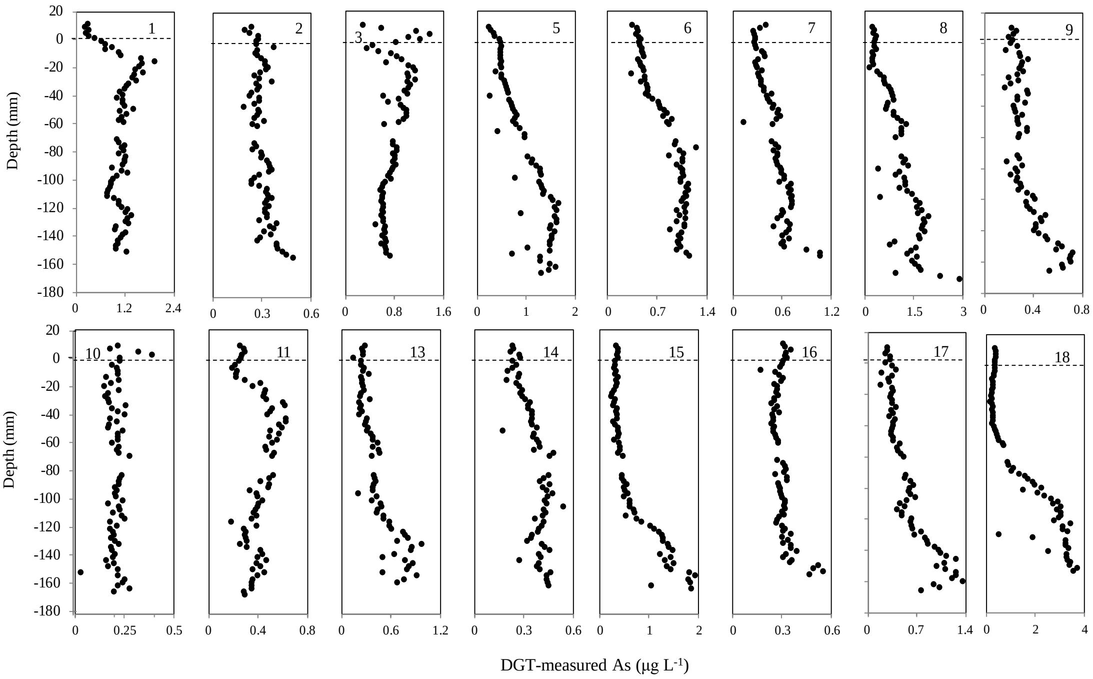

Figure S6. The DGT measured As concentrations of sampling sites in Lake Hongze.

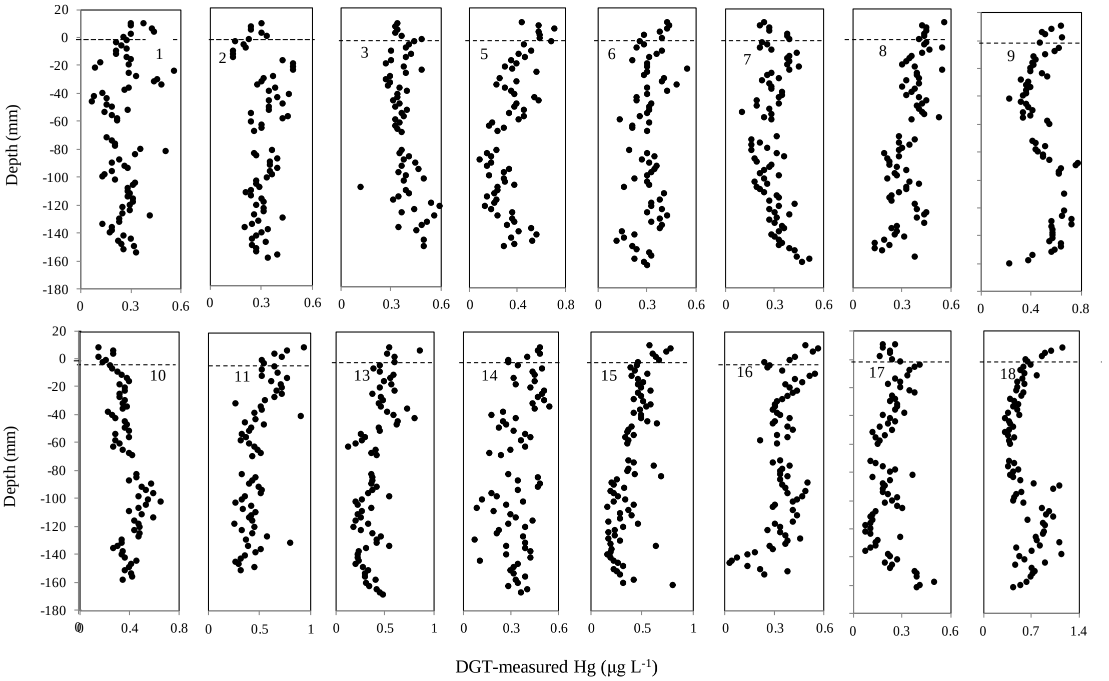

Figure S7. The DGT measured Hg concentrations of sampling sites in Lake Hongze.

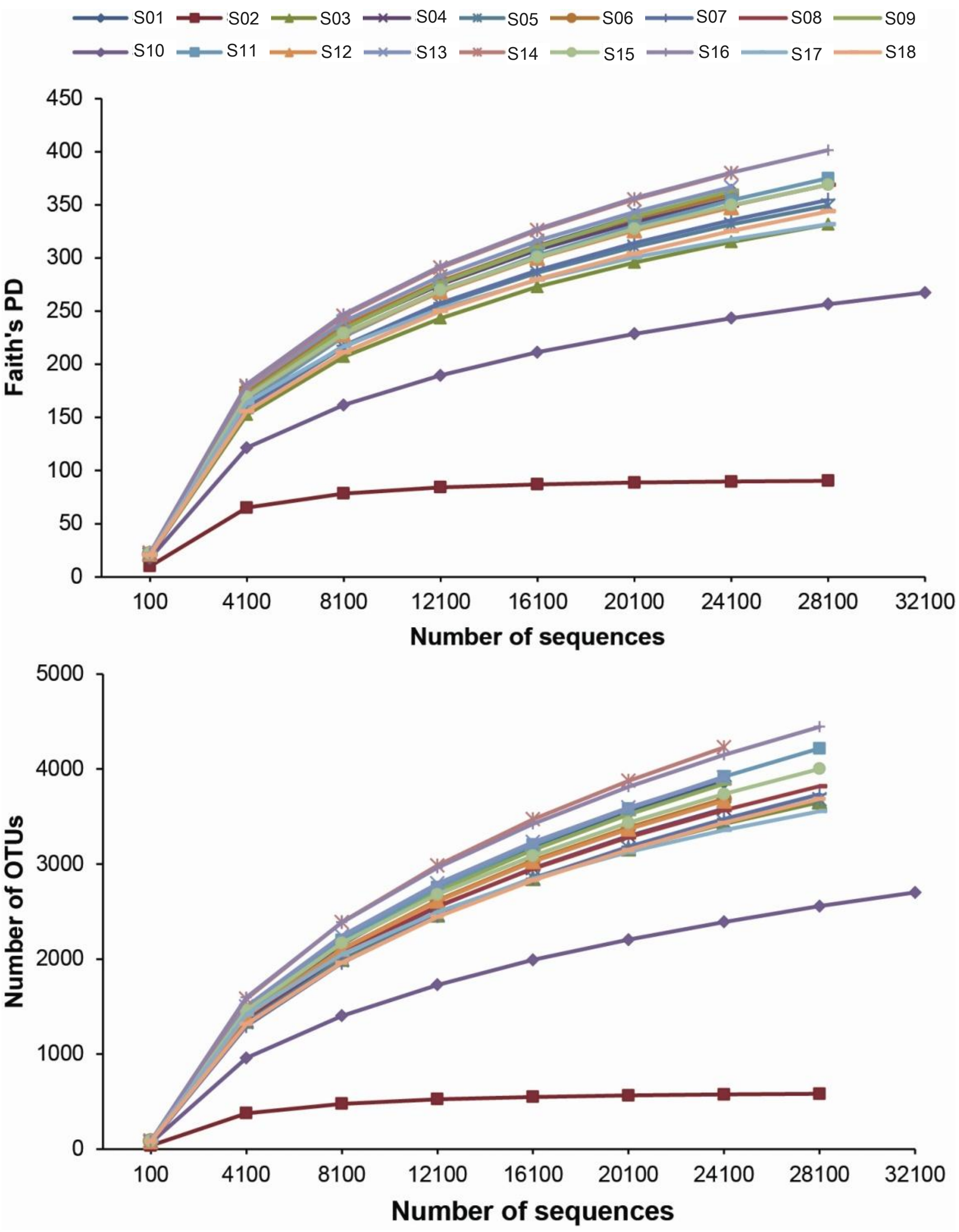

Figure S8. The rarefaction curves of sampling sites in Lake Hongze.

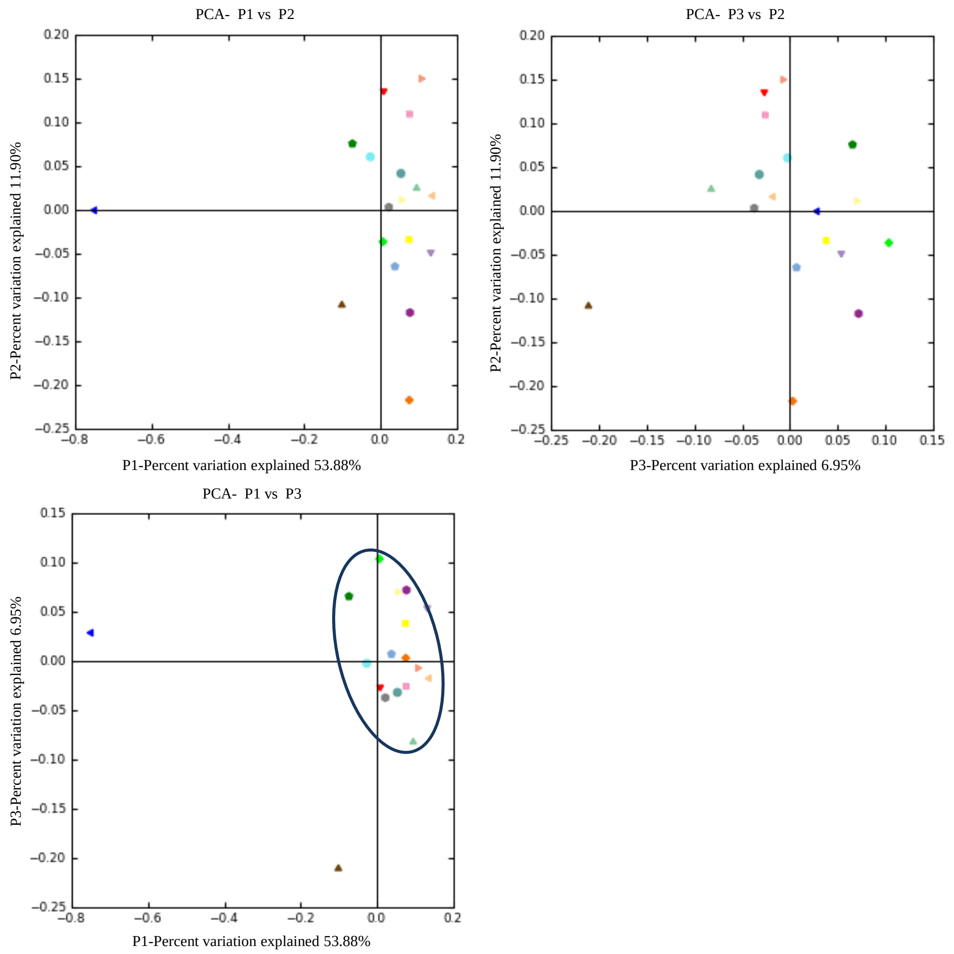

Figure S9. The Bray–Curtis of sampling sites in Lake Hongze.

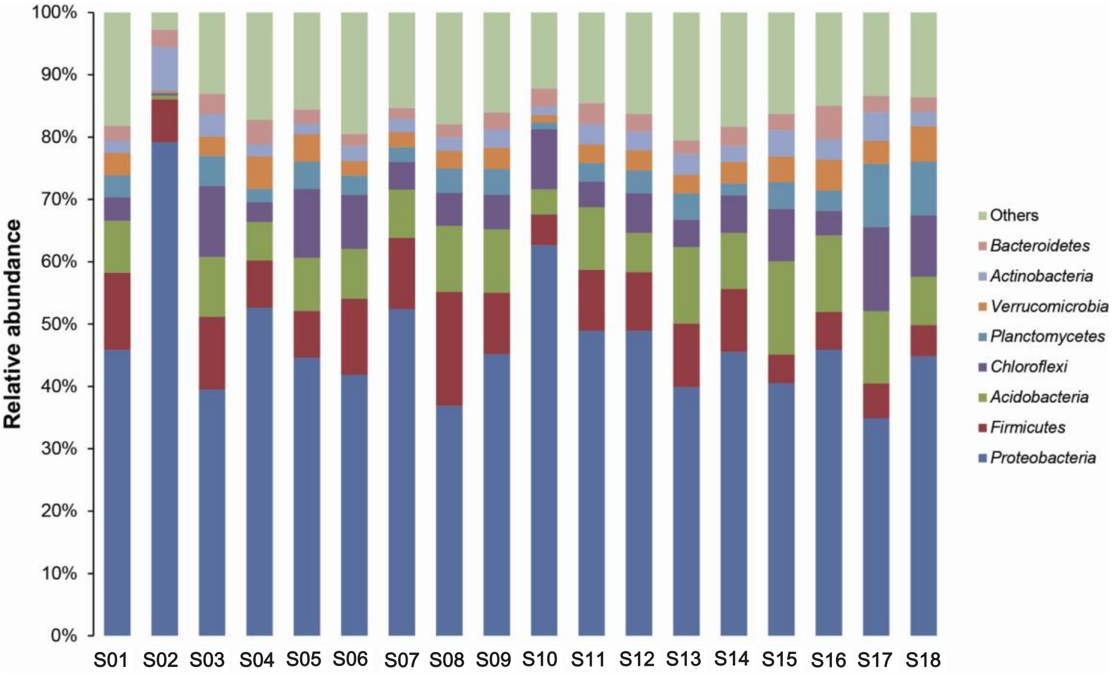

Figure S10. The relative abundance of the bacteria and archaea at Phylum in sampling sites of Lake Hongze.

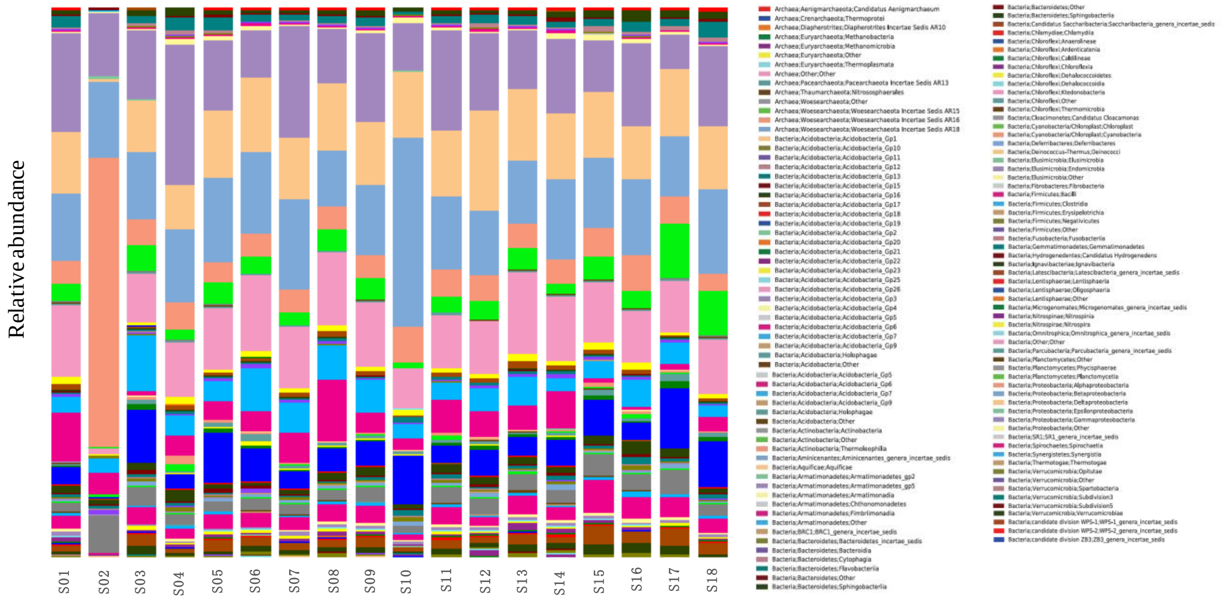

Figure S11. The relative abundance of the bacteria and archaea at class level in sampling sites of Lake Hongze.

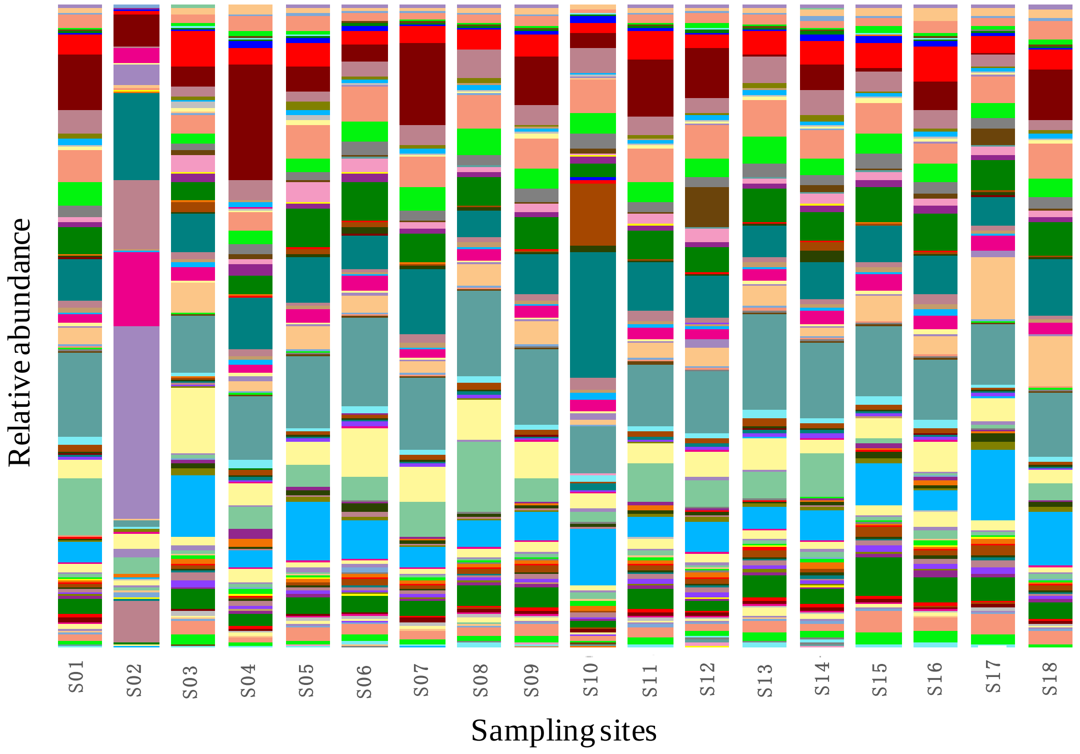

Figure S12. The relative abundance of the bacteria and archaea at gene level in sampling sites of Lake Hongze. The detailed legends are listed in Supplementary Information 2.

Table S1. Average mass in blank resin and measurement of detection limit of Hg, As, Fe, Mg, Cd, Pb and Mn.

	**Hg**	**As**	**Fe**	**Mg**	**Cd**	**Pb**	**Mn**
DGT	3-Mercaptopropyl functionalized silica gel	ZrO	Chelex-100	Chelex-100	Chelex-100	Chelex-100	Chelex-100
MBG ($ng\cdot cm^{-2}$)	0.37 ± 0.11	4.2 ± 0.43	20.58 ± 0.77	7.9 ± 0.16	6.1 ± 0.51	3.9 ± 0.43	31.2 ± 0.79
MDL ($\mu g\cdot L^{-1}$)	0.018	0.204	0.73	1.002	0.384	0.297	0.846
